# Supplementary material for: Awareness of and positive attitudes toward HIV pre-exposure prophylaxis among reproductive-aged women in Ghana: A multilevel analysis of the 2022 Demographic and Health Survey
Source: PLoS One. 2026 May 12;21(5):e0347111. doi: 10.1371/journal.pone.0347111 (PMC13166956; doi:10.1371/journal.pone.0347111)
Supplement: Attachment — Submitted filename: Reviewer comments_PONE R1_09022026.pdf [file pone.0347111.s002.pdf]

Review date February 09, 2026

Journal PLOS ONE

Manuscript ID PONE-D-25-54678R1

Awareness of HIV Pre-Exposure Prophylaxis and Positive Attitudes among Reproductive-Aged Women in Ghana: A Multilevel Analysis of the 2022 Demographic and Health Survey

Reviewer's report

This study examined the factors associated with PrEP awareness and positive attitudes toward its use among women of reproductive age in Ghana. The authors reported that, overall, 12.13% of women were aware of PrEP and had positive attitudes toward its use. The Ashanti region had the highest proportion, 20.25%, whereas the Northern region had the lowest. The main predictors of PrEP awareness and positive attitudes toward its use included being aged 25–29 years, 30–39 years, and 40–44 years; having heard about STIs; reporting higher education; listening to the radio; and residing in the Ahafo region, Oti region, and Upper East region. However, those with ≥ two sexual partners had lower awareness and positive attitudes toward PrEP.

This revised manuscript has been improved substantially in terms of wording consistency and clarity. But I still have fewer questions and comments that authors need to further address and specify throughout the manuscript before it could be considered for publication. Here are the questions, comments and specifications that could be additionally addressed or revised to improve the clarity and content of the manuscript.

Abstract

1- Results**,** please recheck sentences and figures with **Table 1** for consistency. Authors should be clearer such as *"...The Ashanti region had the highest proportion of sample distribution, 20.24%, whereas the North–East had the lowest, 1.68%"* rather than *"...The Ashanti region had the highest proportion, 20.25%, whereas the Northern had the lowest, 1.68%".*

Background

2- Paragraph: Line 118-123: It should add few citations about HIV PrEP awareness and positive attitudes toward its use in different regions such as Asia, SSA among women rather than citing among the MSM and sex worker.

Methods

3- Line 146-147 "*The Ghana Health Service Ethics Review Committee approved the study*" should be moved to Ethical consideration section.

4- It is not clear to me about outcome variable, could the authors detail more such as "How many questions are asked about awareness and how many about attitudes?" Any cut-off point defined as awareness and having positive attitudes toward PrEP?

5- Explanatory variables: Could you specify what variables were included in an individual level and what variables were included in a cluster level or community level since you used the multilevel analysis?

Results

6- The region variable was divided into 17 geographical regions. Is it a nationally pre-defined region? If not, some regions should be combined together due to uneven disproportionate distributions, such as the Ashanti region, which has a large sample distribution (20.24%), compared to Savannah (1.85%), North-East (1.68%) and others.

References

7- It would be better to shorten numbers of reference citations down to about 35 or less since it is too much references (56) for such an research article.

I declare that I have no conflicting interest in reviewing this manuscript.
