# Supplementary material for: Awareness of and positive attitudes toward HIV pre-exposure prophylaxis among reproductive-aged women in Ghana: A multilevel analysis of the 2022 Demographic and Health Survey
Source: PLoS One. 2026 May 12;21(5):e0347111. doi: 10.1371/journal.pone.0347111 (PMC13166956; doi:10.1371/journal.pone.0347111)
Supplement: Attachment — Submitted filename: Comment.docx [file pone.0347111.s001.docx]

Comment

Well written manuscript on Knowledge and attitudes toward HIV Pre-Exposure prophylaxis among reproductive aged women in Ghana: A Multilevel Analysis of the 2022 Demographic and Health Survey.

A few comments:

Abstract

1. It is important to describe in the methods how the questions explored knowledge and attitude. Who is knowledgeable? What do you mean by attitude (good/bad?). One/two sentences on how these variables were measured should suffice
2. Report higher or lower odds of outcome. Odds ratio is not a risk ratio. Do not use word like more likely/less likely, likelihood when using Odds ratio. These terminologies should be used when interpreting Risk ratio, Hazard Ratio, and Incidence Rate Ratio. This comment should be cross checked throughout the manuscript.
3. Up until reading the conclusion, results start making sense when you say positive attitude .Otherwise it was not flowing well when you say have knowledge and attitude. It should in fact be good knowledge and positive attitude if you like. Please make correction throughout the abstract.

Main text

1. Would you please share the reason(s) of using multilevel modelling in the analysis section?
2. Is wealth index a community variable? It does not change results, but I am just concerned treating it as community variable. I would understand place of residence and regions where participants are nested.
3. Please edit title of figure 1: Is it PrEP awareness prevalence? PrEP awareness and positive attitude prevalence. It should be clear
4. Line 81, define MSM, it seems that it is the first time it appears in the main text
5. Line 255, 258 etc. Once defined and abbreviated, use abbreviations throughout. I see PrEP being defined again and MSM was used earlier but now defined.
6. Line 258-259: Your study was among reproductive aged women from general population. It would be better to compare results with studies of similar population instead of using MSM, TGW as comparison groups.
