# Supplementary material for: Awareness of and positive attitudes toward HIV pre-exposure prophylaxis among reproductive-aged women in Ghana: A multilevel analysis of the 2022 Demographic and Health Survey
Source: PLoS One. 2026 May 12;21(5):e0347111. doi: 10.1371/journal.pone.0347111 (PMC13166956; doi:10.1371/journal.pone.0347111)
Supplement: Attachment — Submitted filename: Response to Reviewers Comments on PrEP_R2.docx [file pone.0347111.s003.docx]

1

Review date February 09, 2026

Journal PLOS ONE

Manuscript ID PONE-D-25-54678R1

**Awareness of HIV Pre-Exposure Prophylaxis and Positive Attitudes among Reproductive-Aged Women in Ghana: A Multilevel Analysis of the 2022 Demographic and Health Survey**

**Reviewer's report**

This study examined the factors associated with PrEP awareness and positive attitudes toward its use among women of reproductive age in Ghana. The authors reported that, overall, 12.13% of women were aware of PrEP and had positive attitudes toward its use. The Ashanti region had the highest proportion, 20.25%, whereas the Northern region had the lowest. The main predictors of PrEP awareness and positive attitudes toward its use included being aged 25–29 years, 30–39 years, and 40–44 years; having heard about STIs; reporting higher education; listening to the radio; and residing in the Ahafo region, Oti region, and Upper East region. However, those with ≥ two sexual partners had lower awareness and positive attitudes toward PrEP.

This revised manuscript has been improved substantially in terms of wording consistency and clarity. But I still have fewer questions and comments that authors need to further address and specify throughout the manuscript before it could be considered for publication. Here are the questions, comments and specifications that could be additionally addressed or revised to improve the clarity and content of the manuscript.

**Abstract**

1 - Results**,** please recheck sentences and figures with **Table 1** for consistency. Authors should be clearer such as *“...The Ashanti region had the highest proportion of sample distribution, 20.24%, whereas the North‒East had the lowest, 1.68%”* rather than *“...The Ashanti region had the highest proportion, 20.25%, whereas the Northern had the lowest, 1.68%”.*

Response:

Thank you for this observation. The proportions reported refer to **PrEP awareness and positive attitudes**, which correspond to **Table 2 rather than Table 1**. However, we agree that the values reported in the abstract were inconsistent with the table. The abstract has now been revised to ensure that all figures correspond exactly with those reported in Table 2.

**Background**

1 - Paragraph: Line 118-123: It should add few citations about HIV PrEP awareness and positive attitudes toward its use in different regions such as Asia, SSA among women rather than citing among the MSM and sex worker.

Response: Thank you for this helpful suggestion. We revised the background section to include studies on PrEP awareness and positive attitudes among women and general populations in sub-Saharan Africa, specifically citing evidence from a multi-country study among women and a population-based study from Malawi [21,22]. We retained the Ghana studies among MSM and FSWs to provide local context, while clarifying that little is known about PrEP awareness and positive attitudes among women in the general population in Ghana.

**Methods**

1 - Line 146-147 “*The Ghana Health Service Ethics Review Committee approved the study*” should be moved to Ethical consideration section.

Response: Thank you for this observation. We have moved the statement “The Ghana Health Service Ethics Review Committee approved the study” from the Study design and description section to the Ethical consideration section for better organisation and clarity.

1 - It is not clear to me about outcome variable, could the authors detail more such as “How many questions are asked about awareness and how many about attitudes?” Any cut-off point defined as awareness and having positive attitudes toward PrEP?

Response: Thank you for this important comment. We have revised the description of the outcome variable in the Methods section to provide more clarity. Specifically, PrEP awareness and attitudes were assessed using questions from the 2022 Ghana Demographic and Health Survey (GDHS) women’s questionnaire. Respondents were first asked whether they had ever heard of HIV pre-exposure prophylaxis (PrEP). Those who answered “yes” were asked a follow-up question regarding whether they approved of people taking PrEP to prevent HIV infection.

To construct the outcome variable, we created a composite binary measure. Respondents who reported that they had heard of PrEP and approved of people taking PrEP to prevent HIV infection were classified as having PrEP awareness and a positive attitude (coded = 1). All other responses, including those who had never heard of PrEP, did not approve of its use, or were unsure, were coded as 0. The Methods section has been revised accordingly to clarify the number of questions used and the cut-off criteria applied in the analysis.

2 -
3 - Explanatory variables: Could you specify what variables were included in an individual level and what variables were included in a cluster level or community level since you used the multilevel analysis?

Response:

Thank you for this helpful comment. We have revised the Methods section to clearly distinguish between the individual-level and contextual (household and community-level) variables included in the multilevel analysis. Individual-level variables included women’s age, educational attainment, marital status, religion, employment status, exposure to mass media (reading newspaper or magazine, listening to radio, watching television, and internet use), number of sexual partners excluding spouse, awareness of other sexually transmitted infections (STIs), and whether the respondent visited a health facility in the past 12 months. The contextual-level variables included the wealth index, place of residence (urban/rural), and region. These clarifications have now been added to the “Explanatory variables” subsection of the Methods section.

**Results**

1 - The region variable was divided into 17 geographical regions. Is it a nationally pre-defined region? If not, some regions should be combined together due to uneven disproportionate distributions, such as the Ashanti region, which has a large sample distribution (20.24%), compared to Savannah (1.85%), North-East (1.68%) and others.

Response:

Thank you for this valuable comment. We would like to clarify that the regional variable used in this study reflects the 16 officially recognised administrative regions of Ghana, as defined following the 2019 regional reorganisation and captured in the 2022 Ghana Demographic and Health Survey (GDHS). These regions are nationally predefined administrative units and form part of the DHS sampling framework. The unequal distribution of respondents across regions reflects differences in population size and sampling allocation across the country. However, the DHS employs a complex sampling design and sampling weights to ensure national representativeness. These weights were applied in our analysis to account for disproportionate sampling. Additionally, retaining the official regional categories allows for more meaningful geographic interpretation of the findings and supports policy-relevant recommendations. For these reasons, we retained the 16 administrative regions as separate categories rather than combining them.

**References**

1 - It would be better to shorten numbers of reference citations down to about 35 or less since it is too much references (56) for such an research article.

Response:

Thank you for this helpful suggestion. We carefully reviewed the reference list and ensured that all cited studies are directly relevant to the background, methodology, and discussion of the study. Given the need to provide adequate context on HIV prevention and PrEP research in sub-Saharan Africa and Ghana, we retained the references that are essential to support the arguments presented in the manuscript. However, we have checked the list to remove any redundant citations where possible.

I declare that I have no conflicting interest in reviewing this manuscript.
